# Evaluation of the Efficacy of Methyl Bromide in the Decontamination of Building and Interior Materials Contaminated with Bacillus anthracis Spores

**DOI:** 10.1128/AEM.03445-15

**Published:** 2016-03-21

**Authors:** Joseph P. Wood, Morgan Wendling, William Richter, Andrew Lastivka, Leroy Mickelsen

**Affiliations:** a U.S. Environmental Protection Agency, Office of Research and Development, National Homeland Security Research Center, Research Triangle Park, North Carolina, USA; b Battelle Memorial Institute, West Jefferson, Ohio, USA; c U.S. Environmental Protection Agency, Office of Emergency Management, Research Triangle Park, North Carolina, USA; Rutgers, The State University of New Jersey

## Abstract

The primary goal of this study was to determine the conditions required for the effective inactivation of Bacillus anthracis spores on materials by using methyl bromide (MeBr) gas. Another objective was to obtain comparative decontamination efficacy data with three avirulent microorganisms to assess their potential for use as surrogates for B. anthracis Ames. Decontamination tests were conducted with spores of B. anthracis Ames and Geobacillus stearothermophilus, B. anthracis NNR1Δ1, and B. anthracis Sterne inoculated onto six different materials. Experimental variables included temperature, relative humidity (RH), MeBr concentration, and contact time. MeBr was found to be an effective decontaminant under a number of conditions. This study highlights the important role that RH has when fumigation is performed with MeBr. There were no tests in which a ≥6-log_10_ reduction (LR) of B. anthracis Ames was achieved on all materials when fumigation was done at 45% RH. At 75% RH, an increase in the temperature, the MeBr concentration, or contact time generally improved the efficacy of fumigation with MeBr. This study provides new information for the effective use of MeBr at temperatures and RH levels lower than those that have been recommended previously. The study also provides data to assist with the selection of an avirulent surrogate for B. anthracis Ames spores when additional tests with MeBr are conducted.

## INTRODUCTION

In 2001, at least five envelopes containing virulent Bacillus anthracis (Ames strain) spores were sent through the United States Postal Service to various locations in Florida, New Jersey, New York State, and the Washington, DC, area. At least 22 victims contracted anthrax disease as a result of the mailings, resulting in 5 victim fatalities. Postal facilities in Trenton, NJ, and Washington, DC, were heavily contaminated with B. anthracis (Ames) spores and underwent extensive remediation efforts (1, 2). The overall cost of the environmental remediation across the United States in response to the 2001 release of B. anthracis spores (referred to as the Amerithrax attack) is estimated to have been about $320 million (3). The use of sporicidal chemicals to completely decontaminate (essentially sterilize) such large buildings was unprecedented (4).

In the event of a large urban release of B. anthracis spores, extensive resources would be required in the recovery effort, and the number of private decontamination contractors available may not be sufficient to respond to the decontamination needs (5). Methyl bromide (MeBr) has been identified to be an option for the decontamination of large indoor and outdoor civilian areas following such an aerosol release of B. anthracis spores over a wide urban area (6). Although the production and importation of MeBr are being phased out, in compliance with the *Montreal Protocol on Substances That Deplete the Ozone Layer* (7), there are approximately 3.2 million kg of MeBr still used annually in the United States under certain exemptions. These include the quarantine and preshipment exemption, to eliminate quarantine pests, and the critical use exemption, designed for agricultural users with no technically or economically feasible alternatives. MeBr has other advantages as a decontaminant: there are a large number of personnel trained in its use, it easily penetrates materials, and it is relatively compatible with (does not cause damage to) most materials.

MeBr has been demonstrated via a limited number of laboratory tests to have sporicidal activity against spores of B. anthracis (the bacterium causing anthrax disease) and other spore-forming species (8–11). However, these tests were predominantly conducted at a relatively high temperature (e.g., 37°C) or a high relative humidity (RH) level (75%), had other limitations (e.g., they had minimal bacterial spore loadings; they tested too few materials; and MeBr concentrations, temperatures, or RH levels were unreported), or insufficiently described the methods used. In contrast, the present study focused primarily on determination of the conditions for effectively inactivating B. anthracis Ames spores when using MeBr at relatively low temperatures and RHs, coupled with longer contact times, and using several relevant building and outdoor materials. The demonstration of successful decontamination with MeBr gas at temperatures and RH levels lower than those that have been used previously would facilitate its use, which would be critical should large-scale decontamination efforts be required in the event of a wide B. anthracis release.

Another objective of the study was to obtain side-by-side inactivation efficacy data by comparison of the inactivation efficacy of B. anthracis Ames with that of three avirulent spore-forming microorganisms, to assess their potential for use as representative surrogates for B. anthracis Ames in future decontamination studies with MeBr. Previous tests with Bacillus atrophaeus showed this species to be excessively resistant to MeBr compared to the resistance of virulent B. anthracis (Ames) (11), and so it was not included in the present study. The Ames strain of B. anthracis was chosen for testing in the present study because it was the strain identified in the Amerithrax incident in 2001 (12).

## MATERIALS AND METHODS

### Test organisms.

Spores of virulent B. anthracis (Ames strain) were prepared using a BioFlo 3000 fermentor (New Brunswick Scientific Co., Inc., Edison, NJ, USA) as previously described (13, 14). Briefly, a primary culture of B. anthracis Ames was grown overnight (16 to 18 h at 37°C) in nutrient broth (BD Diagnostic Systems, Franklin Lakes, NJ, USA) on an orbital shaker set at 200 rpm. This primary culture was used to inoculate a scale-up culture that was grown for 6 to 8 h in nutrient broth at 200 rpm. The scale-up culture was then used to inoculate Leighton-Doi broth (BD Diagnostic Systems) in the fermentor. Cultures were grown in the fermentor for approximately 24 h at 37°C. Cultures exhibiting >80% refractile spores, as determined by phase-contrast microscopy (model DC500; Leica Microsystems, Bannockburn, IL, USA), were centrifuged at approximately 10,000 to 12,000 × *g* for 15 to 20 min at 2 to 8°C. The resultant pellet was washed twice and resuspended in ice-cold, sterile water. The suspension was heat shocked by incubation at 60°C for 45 to 60 min to kill vegetative cells, centrifuged, and washed a minimum of two times in ice-cold, sterile water to remove cellular debris. The spore preparation was purified by centrifuging the sample through a gradient of ice-cold, sterile 58% Hypaque-76 (Nycomed Amersham, Princeton, NJ, USA) at 9,000 × *g* for 2 h at 2 to 8°C.

Geobacillus stearothermophilus (ATCC 12980), B. anthracis NNR1Δ1 (received from Edgewood Chemical and Biological Center, Edgewood, MD), and B. anthracis Sterne 34f2 (Colorado Serum Company, Denver, CO) avirulent spores were tested alongside the virulent form of B. anthracis (Ames) to assess their potential for use as surrogates. The microorganism G. stearothermophilus was selected for testing since this species was used in a previous study with MeBr (10). Spore production techniques generally followed standard methods (15). Using growth from a stock culture, G. stearothermophilus, B. anthracis NNR1Δ1, or B. anthracis Sterne was inoculated into 10-ml tubes of nutrient broth and incubated in a shaking incubator for 24 ± 2 h with shaking at approximately 150 rpm. The incubation temperature for the B. anthracis strains was 37°C, while for G. stearothermophilus, an incubation temperature of 55°C was used. This culture was used to inoculate amended nutrient agar plates. Plates were inoculated with 500 μl of the culture, and the inoculum was spread with a sterile plate spreader. The plates were inverted (with no shaking) and incubated for 12 to 14 days. Following incubation, the growth on the plates was harvested by washing with 10 ml sterile water and scraped into sterile tubes. The harvested spores from the B. anthracis strains and G. stearothermophilus were centrifuged at 5,000 rpm, washed with water three times, and resuspended in sterile water. The prepared spores were examined via microscopy, and the preparations were determined to have >95% refractile spores with <5% cellular debris. All stock spore suspensions were prepared in sterile water at an approximate concentration of 10^9^ CFU/ml and stored under refrigeration at 2 to 8°C.

### Coupon materials and related procedures.

In general, test coupons were prepared as previously described (13); coupons were 1.9 cm by 7.5 cm and sterilized prior to testing. Coupons of flat glass (Brooks Brothers Glass & Mirror, Columbus, OH, USA) and unpainted concrete (C90 cinder block; Wellnitz Concrete, Columbus OH) were sterilized by autoclaving. Coupons of industrial-grade carpet (Shaw Industries, Dalton, GA), ceiling tile (B513; Armstrong, Columbus, OH), painted wallboard paper (United States Gypsum Company, Chicago, IL, USA), and bare pine wood (generic wood molding; Lowes, Columbus, OH) were sterilized via gamma irradiation at 40 kGy (Steris Isomedix Services, Libertyville, IL).

Test and positive-control coupons were placed on a flat surface within a class II biological safety cabinet (BSC) and inoculated with a target quantity of 1 × 10^8^ CFU of viable B. anthracis Ames (or surrogate) spores per coupon. A 100-μl aliquot of a stock suspension of approximately 1 × 10^9^ CFU/ml was dispensed using a micropipette and was applied as 10 10-μl droplets across the coupon surface. The actual titer of the inoculum was verified for each test using standard dilution plating techniques (discussed in more detail below). After inoculation, the coupons were transferred to a class III BSC and left undisturbed overnight to dry under ambient conditions (approximately 22°C and 40% RH).

The number and type of replicate coupons used for each test were as follows: five test coupons inoculated with B. anthracis Ames or surrogate spores and exposed to MeBr, five positive controls inoculated with B. anthracis Ames or surrogate spores but not exposed to MeBr, one laboratory blank not inoculated and not exposed to MeBr, and one procedural blank not inoculated and exposed to MeBr. On the day following liquid spore inoculation, test coupons intended for decontamination, including blanks, were transferred into a test chamber and exposed to the MeBr. Positive-control coupons were transferred to the positive-control chamber (discussed below).

### MeBr fumigation procedures and test matrix.

MeBr (Great Lakes Chemical Corporation, West Lafayette, IN) is a colorless and odorless volatile gas. Chloropicrin was added to the MeBr source gas (0.5% chloropicrin, 99.5% MeBr) as a warning irritant (lacrimator) for the safety of laboratory staff. The gas mixture was used at full strength and injected into the test chamber to achieve the indicated target concentration.

Decontamination tests were conducted inside a 38-liter stainless steel chamber. The chamber was insulated to prevent condensation on the inside chamber walls. As a means of secondary containment and for the safety of the laboratory personnel, this test chamber was housed inside a custom acrylic compact glove box (Plas Labs, Inc., Lansing, MI) that was hard ducted to the facility exhaust system. The coupons were placed on wire racks inside the chamber. A small mixing fan was laced inside the chamber to maintain homogeneous gas conditions.

The air temperature was controlled using a small heat exchanger connected to a heated/cooled water bath, and RH was elevated as needed using a Nafion tube pervaporation system (which was controlled using a water bath). The temperature and RH in the test chamber were measured using an HMT368 temperature and humidity probe (Vaisala, Inc., Woburn, MA). The temperature, RH, and MeBr concentration were controlled with a CNI-822 controller (Omega Engineering, Stamford, CT), and data were recorded every minute during the contact time using the associated iLOG software.

The MeBr concentration in the test chamber was measured continuously during the contact period using a Fumiscope meter (version 5.0; Key Chemical and Equipment Company, Clearwater, FL). MeBr was added to the chamber, as necessary, to maintain the specified concentration within ±10%. The Fumiscope meter was calibrated by the manufacturer and displays the concentration (in ounces) of MeBr per 1,000 cubic feet (approximately 1 mg/liter, independent of temperature). The Fumiscope meter included an air pump that pulled a gas sample from the test chamber through the thermal conductivity meter at a controlled rate and exhausted the gas back into the test chamber.

A 9-liter Lock & Lock airtight container (Lock & Lock, Farmers Branch, TX) served as the positive-control test chamber. Fixed-humidity-point salts were added as a slurry to a separate container placed in the bottom of the MeBr positive-control chamber. Sodium chloride was used to control the RH at 75%, and potassium carbonate was used to control the RH at 45%. The control chamber was placed in an incubator (Thermo Scientific, Waltham, MA) for all tests and set to the appropriate temperature (i.e., 22, 27, or 32°C). The temperature and RH of the positive-control chamber were measured, and data were logged using a Hobo data logger (model U12-11; Onset Computer Corporation, Cape Cod, MA).

Twenty tests were conducted with MeBr at a concentration of either 212 or 300 mg/liter, as shown in Table 1. Each test used B. anthracis Ames and at least one surrogate species or strain. During each test run, inoculated test samples were placed inside the MeBr test chamber, and the chamber was sealed. The chamber was allowed sufficient time to equilibrate to the target temperature and RH prior to the start of the run. Once the temperature and RH were stable, MeBr was slowly injected into the chamber until the target concentration was reached. The test chamber remained sealed until the end of the required contact time. At this time, the valve to the MeBr source was turned off and the seal of the test chamber was broken by removing the lid. The test chamber and the class III BSC were allowed to aerate until the MeBr levels in the chamber reach 0 mg/liter, which happened within minutes of lid removal. At this time, the test samples were removed and processed as described below. Positive controls were also then processed at this time.

**TABLE 1:** Test matrix^*a*^

Test no.	Surrogate microorganism(s)	Target fumigation parameter value			Contact time (h)
		MeBr concn (mg/liter)	Temp (°C)	RH (%)	
1	G. stearothermophilus	212	22	45	36
2	G. stearothermophilus	212	22	45	48
3	B. anthracis NNR1Δ1	212	22	75	36
4	B. anthracis NNR1Δ1	212	27	45	36
5	B. anthracis NNR1Δ1	212	22	75	24
6	B. anthracis Sterne	212	27	45	48
7	B. anthracis Sterne	212	27	75	24
8	B. anthracis Sterne	212	27	45	48
9	B. anthracis NNR1Δ1, B. anthracis Sterne	212	27	75	36
10	B. anthracis NNR1Δ1, B. anthracis Sterne	300	22	45	48
11	B. anthracis NNR1Δ1, B. anthracis Sterne	212	22	45	60
12	B. anthracis NNR1Δ1, B. anthracis Sterne	212	32	75	24
13	B. anthracis NNR1Δ1, B. anthracis Sterne	212	32	45	48
14	B. anthracis NNR1Δ1, B. anthracis Sterne	300	22	75	24
15	B. anthracis NNR1Δ1, B. anthracis Sterne	300	22	45	60
16	B. anthracis NNR1Δ1, B. anthracis Sterne	212	32	45	60
17	B. anthracis NNR1Δ1, B. anthracis Sterne	300	27	75	18
18	B. anthracis NNR1Δ1, B. anthracis Sterne	300	27	45	60
19	B. anthracis NNR1Δ1, B. anthracis Sterne	212	32	45	72
20	B. anthracis NNR1Δ1, B. anthracis Sterne	300	32	45	60

a All tests were conducted with B. anthracis Ames and at least one surrogate. Tests 1 to 8 were conducted with glass, ceiling tile, carpet, painted wallboard paper, bare pine wood, and unpainted concrete. Tests 9 to 20 were conducted with glass, ceiling tile, carpet, and bare pine wood.

As the investigation proceeded, adjustments were made to one of the fumigation parameters (contact time, temperature, RH, MeBr concentration) to assess the effect of that parameter and to find conditions that were efficacious with the least contact time. The first eight tests were conducted with all six materials and B. anthracis Ames and one other species. In tests 9 to 20, two materials were eliminated from testing (unpainted concrete and painted wallboard paper) to allow the simultaneous testing of three microorganisms. In the latter 12 tests, testing focused on the B. anthracis strains of Ames, Sterne, and NNR1Δ1 and G. stearothermophilus was no longer tested. Unpainted concrete and painted wallboard paper were removed from the latter phase of testing as the decontamination efficacy was the highest for these materials. Tests 6 and 8 utilized the same operational parameters to assess repeatability.

### Spore recovery from coupons and quantification.

To extract spores from materials, the test coupons, positive controls, and blanks were placed in 50-ml polypropylene conical tubes containing 10 ml of sterile phosphate-buffered saline (PBS; 99.9%; Sigma, St. Louis, MO, USA) and 0.1% Triton X-100 (Sigma) (PBST). The vials were capped, placed on their sides, and agitated on an orbital shaker for 15 min at 200 rpm at room temperature. Following agitation, a 1-ml extract was removed and a series of 10-fold dilutions was prepared in sterile water. An aliquot (0.1 ml) of either the undiluted extract or each serial dilution was plated onto tryptic soy agar in triplicate and incubated for 18 to 24 h at the appropriate temperature. Colonies were counted manually, and the numbers of CFU per milliliter were determined by multiplying the average number of colonies per plate by the reciprocal of the dilution. Dilution data, representing the greatest number of individually definable colonies, were expressed as the arithmetic mean of the numbers of CFU observed ± the standard deviation (SD). Laboratory blanks controlled for sterility, and procedural blanks controlled for viable spores inadvertently introduced onto the test coupons. The blanks were spiked with an equivalent amount of 0.1 ml of sterile water. The target acceptance criterion was that extracts of laboratory or procedural blanks were to contain no CFU.

### Recovery and decontamination efficacy calculations.

The recovery of spores from each set of positive controls was calculated for each test with each material and microorganism using the following equation:

(1) $$\text{mean percent recovery}=\left(\text{mean}\;{\text{CFU}}_{\text{pc}}/{\text{CFU}}_{\text{spike}}\right)\times 100$$

where mean CFU_pc_ is the mean number of CFU recovered from the five replicate positive-control (pc) coupons for a given material, and CFU_spike_ is the number of CFU spiked (inoculated) onto each of those coupons, determined by analysis of the inoculum on each day of testing.

The decontamination efficacy of each combination of coupon type and microorganism was calculated in terms of the log_10_ reduction (LR). The number of CFU recovered after each test from each test coupon (CFU_*t*_) and positive-control coupon (CFU_pc_) was transformed to its log_10_ value. Then, the mean of the log_10_ values for each test coupon (5 replicates) was subtracted from the mean of the log_10_ values for each positive control (5 replicates), as follows:

(2) $$\text{efficacy}=\left(\overset{¯}{\log\;{\text{CFU}}_{\text{pc}}}\right)-(\overset{¯}{\log\;{\text{CFU}}_{t}})$$

Test coupons from which no CFU were recovered were assigned a CFU count of 1, resulting in a log CFU of 0. In such cases, the LR is reported as a value greater than or equal to the value calculated by equation 2.

Each of the LR results is reported with an associated 95% confidence interval (CI), calculated as follows:

(3) 95%CI=efficacy±(1.96×SE)

The term SE is the pooled standard error and was calculated as follows:

(4) $$\text{SE}=\sqrt{\frac{{S_{\text{pc}}}^{2}}{5}+\frac{{S_{t}}^{2}}{5}}$$

where *S* is the standard deviation of the LR results for either the 5 positive controls (*S*_pc_) or the 5 test coupons (S_t_) for each combination of decontaminant, coupon material, and microorganism tested.

### Statistical analyses.

All statistical analyses were performed using SAS (version 9.3) software (SAS Institute Inc., Cary, NC). Some comparisons, such as percent recovery from the controls by material, employed a *t* test. When needed to address unequal variances, the Satterthwaite adjustment was used. The Wilcoxon rank sum test was applied for other comparisons, e.g., LR by material or species/strain, with differences being estimated with the Hodges-Lehmann estimator. *P* values were calculated on the basis of the results of these statistical tests, and unless noted otherwise, differences in results are reported as significant using a 5% level (*P* < 0.05).

## RESULTS

### Fumigation conditions.

The average levels for all fumigation parameters (temperature, RH, MeBr concentration) for each test were within ±1% of the intended target values (Table 1). Refer to Table S1 in the supplemental material for the actual fumigation values for each test.

### Inoculation levels and recovery from positive controls.

As shown in Table 2, the mean inoculation levels for the overall study for B. anthracis Ames, G. stearothermophilus, B. anthracis NNR1Δ1, and B. anthracis Sterne were 1.09 × 10^8^, 7.29 × 10^7^, 1.13 × 10^8^, and 1.05 × 10^8^ CFU, respectively. The NNR1Δ1 and Sterne inoculation levels were not significantly different from the Ames strain inoculation level. For the first two tests, in which B. anthracis Ames and G. stearothermophilus were used, the inoculation levels for the two species were not significantly different from each other. The average percent recoveries from positive-control coupons for all tests are presented in Table 3. The mean percent recovery from positive controls was significantly higher for the Ames strain than for the other strains, although the estimated differences in percent recovery were less than 7%. Overall, spore recoveries ranged from 0.4% to 87%. With B. anthracis Ames in particular, the lowest recoveries were obtained for bare pine wood (from which the recovery was significantly less than that from all other materials), and the highest recoveries were obtained for carpet (from which the recovery was significantly higher than that from all other materials).

**TABLE 2:** Average inoculum per coupon^*a*^

Strain	Avg inoculum (no. of CFU)/coupon
B. anthracis Ames (*n* = 20)	1.09E+08 ± 2.09E+07
G. stearothermophilus (*n* = 2)	7.29E+07 ± 6.86E+06
B. anthracis NNR1Δ1 (*n* = 15)	1.13E+08 ± 2.00E+07
B. anthracis Sterne (*n* = 15)	1.05E+08 ± 8.45E+06

a *n*, number of tests. Data are means ± SDs.

**TABLE 3:** Average percent recovery from positive controls

Material	Avg % recovery^*a*^			
	B. anthracis Ames (*n* = 20)	G. stearothermophilus (*n* = 2)	B. anthracis NNR1Δ1 (*n* = 15)	B. anthracis Sterne (*n* = 15)
Glass	53.23 ± 15.22	76.87 ± 2.97	55.49 ± 22.6	13.66 ± 8.29
Ceiling tile	11.98 ± 2.44	0.40 ± 0.32	3.41 ± 1.52	12.35 ± 5.01
Carpet	87.46 ± 17.90	22.38 ± 1.62	70.85 ± 21.2	69.35 ± 15.13
Painted wallboard paper	35.65 ± 15.47	67.79 ± 43.27	42.18 ± 23.8	38.60 ± 4.10
Bare pine wood	7.33 ± 2.60	1.60 ± 0.63	6.86 ± 5.71	9.06 ± 3.05
Unpainted concrete	11.24 ± 7.76	12.42 ± 3.86	13.40 ± 7.09	5.96 ± 2.71

a *n*, number of tests. Data are means ± SDs.

### Decontamination efficacy.

A summary of the decontamination efficacy data, in terms of the means and 95% confidence intervals for the LR, is presented in Table 4 for tests 1 to 8 (with all six materials and B. anthracis Ames and one other species) and in Table 5 for tests 9 to 20 (with four materials and B. anthracis strains Ames, NNR1Δ1, and Sterne). Decontamination efficacy varied widely (from less than 1-LR to complete inactivation, in which the LR is reported as a value greater than or equal to the log number of CFU recovered from the positive controls), depending on the test material, organism/strain, and fumigation condition. In most cases, efficacy improved with increasing MeBr concentration, temperature, RH, and contact time. In the first two tests, G. stearothermophilus was inactivated to a higher degree than the B. anthracis Ames strain (or both species were completely inactivated) for every material, and so G. stearothermophilus was eliminated from further testing. We discontinued the use of G. stearothermophilus because for any particular decontaminant, the surrogate microorganism should possess resistance equivalent to or greater than that of the virulent species (13). The G. stearothermophilus population was completely inactivated for nearly every material tested in the first two tests in which it was used. In addition, to allow the simultaneous testing of the three B. anthracis strains (Ames, NNR1Δ1, Sterne), commencing with test 9, we eliminated painted wallboard paper and unpainted concrete from further testing. These two materials generally exhibited the highest LR of the Ames strain in the first eight tests, and their elimination from the test matrix allowed us to focus on the more-difficult-to-decontaminate materials.

**TABLE 4:** Decontamination efficacy results for tests with six materials and B. anthracis Ames and one surrogate

Test no.	Surrogate	Target parameter value		Material	Decontamination efficacy (mean ± 95% CI of log reduction)^*a*^	
		Concn (mg/liter)/contact time (h)	Temp (°C)/RH (%)		Ames	Surrogate
1	G. stearothermophilus	212/36	22/45	Glass	3.14 ± 0.30	≥7.72 ± 0.07
				Ceiling tile	3.47 ± 0.11	4.89 ± 0.90
				Carpet	4.18 ± 0.60	6.77 ± 0.75
				Painted wallboard paper	≥7.04 ± 0.08	≥7.82 ± 0.02
				Bare pine wood	2.16 ± 0.34	≥5.75 ± 0.36
				Unpainted concrete	≥6.17 ± 0.25	≥6.78 ± 0.62
2	G. stearothermophilus	212/48	22/45	Glass	3.00 ± 0.20	≥7.76 ± 0.06
				Ceiling tile	≥6.76 ± 0.09	≥5.00 ± 0.29
				Carpet	6.35 ± 1.55	≥7.19 ± 0.14
				Painted wallboard paper	≥7.21 ± 0.12	≥7.43 ± 0.17
				Bare pine wood	2.82 ± 0.44	≥5.37 ± 0.75
				Unpainted concrete	≥6.89 ± 0.11	≥6.85 ± 0.15
3	B. anthracis NNR1Δ1	212/36	22/75	Glass	≥7.92 ± 0.04	0.60 ± 0.20
				Ceiling tile	≥7.07 ± 0.02	1.12 ± 0.31
				Carpet	≥7.93 ± 0.04	1.56 ± 0.14
				Painted wallboard paper	≥7.76 ± 0.04	0.88 ± 0.15
				Bare pine wood	≥7.02 ± 0.06	1.80 ± 0.24
				Unpainted concrete	≥7.51 ± 0.15	1.26 ± 0.34
4	B. anthracis NNR1Δ1	212/36	27/45	Glass	4.86 ± 0.90	1.97 ± 0.21
				Ceiling tile	6.41 ± 1.26	5.57 ± 1.26
				Carpet	≥7.95 ± 0.03	4.89 ± 0.51
				Painted wallboard paper	≥7.50 ± 0.05	≥7.77 ± 0.08
				Bare pine wood	5.93 ± 1.34	5.08 ± 1.19
				Unpainted concrete	≥7.23 ± 0.24	≥6.88 ± 0.18
5	B. anthracis NNR1Δ1	212/24	22/75	Glass	2.54 ± 0.22	0.35 ± 0.06
				Ceiling tile	2.70 ± 0.48	0.80 ± 0.21
				Carpet	3.31 ± 0.78	0.68 ± 0.12
				Painted wallboard paper	2.69 ± 0.30	0.61 ± 0.04
				Bare pine wood	2.99 ± 0.50	0.84 ± 0.19
				Unpainted concrete	3.41 ± 0.56	1.16 ± 0.47
6	B. anthracis Sterne	212/48	27/45	Glass	3.49 ± 0.74	≥6.97 ± 0.15
				Ceiling tile	4.00 ± 0.65	≥7.00 ± 0.06
				Carpet	6.60 ± 1.66	≥7.87 ± 0.02
				Painted wallboard paper	≥7.34 ± 0.14	≥7.58 ± 0.07
				Bare pine wood	1.86 ± 0.19	4.28 ± 1.53
				Unpainted concrete	≥6.84 ± 0.15	≥6.85 ± 0.26
7	B. anthracis Sterne	212/24	27/75	Glass	4.42 ± 0.96	4.17 ± 1.52
				Ceiling tile	≥7.14 ± 0.06	3.53 ± 1.70
				Carpet	7.40 ± 1.24	2.47 ± 0.33
				Painted wallboard paper	≥7.79 ± 0.02	1.86 ± 0.27
				Bare pine wood	6.29 ± 0.99	1.88 ± 0.34
				Unpainted concrete	≥7.17 ± 0.20	3.83 ± 1.54
8	B. anthracis Sterne	212/48	27/45	Glass	2.29 ± 0.10	6.25 ± 1.08
				Ceiling tile	3.29 ± 0.26	3.99 ± 0.69
				Carpet	4.31 ± 0.55	≥7.78 ± 0.07
				Painted wallboard paper	≥7.60 ± 0.05	≥7.56 ± 0.08
				Bare pine wood	2.03 ± 0.32	3.56 ± 0.98
				Unpainted concrete	≥6.94 ± 0.12	≥6.54 ± 0.21

a Results reported with greater than or equal to signs indicate complete inactivation.

**TABLE 5:** Decontamination efficacy results for tests with B. anthracis strains Ames, NNR1Δ1, and Sterne on four materials

Test no.	Target parameter value		Material	Decontamination efficacy (mean ± 95% CI of log reduction)^*a*^		
	Concn (mg/liter)/contact time (h)	Temp (°C)/RH (%)		Ames	NNR1Δ1	Sterne
9	212/36	27/75	Glass	≥7.74 ± 0.10	2.79 ± 0.68	≥7.53 ± 0.23
			Ceiling tile	≥7.07 ± 0.08	≥6.48 ± 0.30	≥7.06 ± 0.06
			Carpet	≥8.04 ± 0.03	≥7.91 ± 0.08	7.11 ± 0.81
			Bare pine wood	≥6.92 ± 0.08	5.26 ± 1.51	6.50 ± 0.60
10	300/48	22/45	Glass	3.11 ± 0.38	0.62 ± 0.06	6.72 ± 0.73
			Ceiling tile	≥7.23 ± 0.04	6.29 ± 1.40	≥7.13 ± 0.07
			Carpet	4.60 ± 0.89	1.65 ± 0.24	7.32 ± 1.07
			Bare pine wood	2.95 ± 0.42	3.13 ± 0.52	4.14 ± 1.65
11	212/60	22/45	Glass	3.90 ± 0.27	0.87 ± 0.11	5.01 ± 1.31
			Ceiling tile	≥7.16 ± 0.06	5.33 ± 1.14	6.75 ± 0.72
			Carpet	3.41 ± 0.47	1.30 ± 0.13	5.25 ± 1.40
			Bare pine wood	3.03 ± 0.52	3.48 ± 0.48	3.35 ± 0.59
12	212/24	32/75	Glass	≥7.61 ± 0.07	1.73 ± 0.19	≥6.89 ± 0.09
			Ceiling tile	≥7.04 ± 0.05	≥6.64 ± 0.07	≥7.07 ± 0.10
			Carpet	≥7.91 ± 0.06	7.25 ± 0.91	≥7.79 ± 0.06
			Bare pine wood	≥6.89 ± 0.10	3.69 ± 0.40	≥6.84 ± 0.12
13	212/48	32/45	Glass	2.28 ± 0.61	0.60 ± 0.11	3.40 ± 0.34
			Ceiling tile	3.93 ± 0.54	2.67 ± 0.36	4.13 ± 0.38
			Carpet	4.65 ± 0.52	1.63 ± 0.16	7.09 ± 0.88
			Bare pine wood	2.47 ± 0.53	2.06 ± 0.29	3.95 ± 0.59
14	300/24	22/75	Glass	≥7.62 ± 0.07	1.03 ± 0.13	2.57 ± 0.30
			Ceiling tile	6.72 ± 1.12	1.36 ± 0.24	1.61 ± 0.25
			Carpet	≥8.02 ± 0.03	0.75 ± 0.17	2.10 ± 0.30
			Bare pine wood	≥7.04 ± 0.09	1.63 ± 0.28	1.72 ± 0.25
15	300/60	22/45	Glass	3.25 ± 0.37	0.87 ± 0.19	≥6.98 ± 0.18
			Ceiling tile	5.36 ± 1.50	5.87 ± 0.93	4.79 ± 1.32
			Carpet	5.30 ± 0.59	1.85 ± 0.33	7.27 ± 0.81
			Bare pine wood	2.38 ± 0.53	3.60 ± 0.33	3.67 ± 0.34
16	212/60	32/45	Glass	3.03 ± 0.59	0.48 ± 0.22	4.21 ± 1.78
			Ceiling tile	4.36 ± 1.31	2.53 ± 0.43	4.63 ± 1.26
			Carpet	5.29 ± 0.71	2.14 ± 0.42	≥7.80 ± 0.04
			Bare pine wood	2.40 ± 0.68	2.24 ± 0.30	4.15 ± 1.43
17	300/18	27/75	Glass	7.58 ± 0.60	2.68 ± 0.53	4.41 ± 0.32
			Ceiling tile	6.29 ± 1.12	2.33 ± 0.45	2.40 ± 0.37
			Carpet	≥8.11 ± 0.04	1.74 ± 0.21	4.38 ± 1.09
			Bare pine wood	≥6.92 ± 0.06	2.30 ± 0.48	3.04 ± 0.58
18	300/60	27/45	Glass	3.01 ± 0.38	0.73 ± 0.08	5.88 ± 0.80
			Ceiling tile	4.36 ± 0.75	3.38 ± 0.33	3.60 ± 0.33
			Carpet	5.28 ± 0.62	1.73 ± 0.14	7.33 ± 0.84
			Bare pine wood	2.71 ± 0.44	2.88 ± 0.42	3.67 ± 1.04
19	212/72	32/45	Glass	2.72 ± 0.31	0.80 ± 0.12	6.10 ± 1.14
			Ceiling tile	5.20 ± 1.65	3.46 ± 0.50	5.27 ± 1.45
			Carpet	6.02 ± 1.13	1.67 ± 0.40	≥7.88 ± 0.05
			Bare pine wood	2.21 ± 0.16	1.98 ± 0.63	3.41 ± 0.64
20	300/60	32/45	Glass	2.84 ± 0.37	0.44 ± 0.14	3.95 ± 0.58
			Ceiling tile	3.60 ± 0.41	3.19 ± 0.43	4.02 ± 0.47
			Carpet	5.82 ± 1.15	1.89 ± 0.42	7.45 ± 0.60
			Bare pine wood	2.27 ± 0.42	2.96 ± 0.34	3.01 ± 0.31

a Results reported with greater than or equal to signs indicate complete inactivation.

The decontamination efficacy results are further summarized in terms of the minimum contact time required (demonstrated via tests) to achieve at least a 6-LR of B. anthracis Ames on all materials for a given fumigation condition (MeBr concentration, temperature, and RH) (Table 6). The 6-LR benchmark is used since a decontaminant that achieves a 6-LR or greater for a particular material is considered an effective sporicidal decontaminant (16). As seen in Table 6, a contact time of 36 h was required to achieve a ≥6-LR of B. anthracis Ames on all materials when fumigation was with MeBr at 212 mg/liter at 22°C and 75% RH. Only 18 h was required to achieve a ≥6-LR on all materials when the MeBr concentration was increased to 300 mg/liter and the temperature was increased to 27°C (at 75% RH). In none of the tests using 45% RH was a 6-LR or greater of the Ames strain achieved on all materials, even at the highest MeBr concentration (300 mg/liter) and with the longest contact time (72 h) tested.

**TABLE 6:** Contact time demonstrated to achieve a 6-LR of B. anthracis Ames on all materials for a given fumigation condition

MeBr concn (mg/liter)	Temp (°C)	RH (%)	Time (h) required for a 6-LR of Ames on all materials^*a*^	Test no. using fumigation condition
212	22	45	>60	1, 2, 11
	22	75	36	3, 5
	27	45	>48	4, 6, 8
	27	75	36	7, 9
	32	45	>72	13, 16, 19
	32	75	24	12
300	22	45	>60	10, 15
	22	75	24	14
	27	45	>60	18
	27	75	18	17
	32	45	>60	20

a Results reported with a greater than sign indicate that a 6-LR was not achieved for all materials at the longest contact time tested.

The average LR values for B. anthracis Ames are summarized by material in Table 7. Painted wallboard paper and unpainted concrete had the highest average LR values in the first eight tests (6.87 and 6.52, respectively; these results are significantly different from each other at the 10% level), although the average LR for carpet was not significantly different from that for these two materials. Glass and bare pine wood were the most difficult materials to decontaminate in the study, with these materials having significantly lower LR values than the other materials (at the 5 to 10% significance level). Overall, the bare pine wood material was significantly more difficult to decontaminate than glass at the 10% significance level.

**TABLE 7:** Summary of B. anthracis Ames average LRs by material type

Material type	Avg LR ± SD	
	Tests 1 to 8	Tests 9 to 20
Glass	3.96 ± 1.83	4.56 ± 2.30
Ceiling tile	5.11 ± 1.91	5.69 ± 1.38
Carpet	6.00 ± 1.83	6.04 ± 1.61
Painted wallboard paper	6.87 ± 1.71	
Bare pine wood	3.89 ± 2.15	4.02 ± 2.18
Unpainted concrete	6.52 ± 1.32	

A summary of the results comparing the average difference in decontamination efficacy between B. anthracis Ames and the avirulent strains (by test) is shown in Table 8. A positive result in Table 8 indicates that the avirulent strain was inactivated to a higher degree than the Ames strain when the results were averaged across the materials tested. Side-by-side testing was first conducted with G. stearothermophilus and B. anthracis Ames (tests 1 and 2). As mentioned above, the results showed that G. stearothermophilus was significantly less resistant than B. anthracis Ames when exposed to the MeBr gas (*P* = 0.013). The Sterne strain was inactivated to a higher degree than Ames in all tests when the RH was 45% (top portion of Table 8) but was inactivated to a lesser extent than Ames when the tests were conducted at 75% RH. Although overall there was no significant difference in efficacy between the Ames and Sterne strains. The NNR1Δ1 strain was tested alongside B. anthracis Ames in tests 3 to 5 and 9 to 20. This organism was significantly more resistant to MeBr than B. anthracis Ames in all tests performed (*P* < 0.0001). Moreover, in some tests the difference in efficacy between B. anthracis Ames and the NNR1Δ1 strain was excessive, e.g., a difference of a 6.33-LR in test 3 and a difference of a 6.16-LR in test 14.

**TABLE 8:** Summary of average differences in decontamination efficacy between B. anthracis Ames and avirulent strains

Test no.	Target RH (%)	Avg difference in efficacy^*a*^		
		G. stearothermophilus	B. anthracis NNR1Δ1	B. anthracis Sterne
1	45	2.26*	—	—
2	45	1.10	—	—
11	45	—	−1.63	0.72
10	45	—	−1.55	1.86
15	45	—	−1.03	1.61
4	45	—	−1.29	—
6	45	—	—	1.74
8	45	—	—	1.54
18	45	—	−1.66	1.28
13	45	—	−1.59	1.31
16	45	—	−1.92	1.43
19	45	—	−2.06	1.63
20	45	—	−1.51	0.98
5	75	—	−2.20*	—
3	75	—	−6.33*	—
14	75	—	−6.16*	−5.35*
7	75	—	—	−3.75*
9	75	—	−1.83	−0.39
17	75	—	−4.96*	−3.67*
12	75	—	−2.54	−0.22

a The results are shown as the average difference in efficacy (log reduction). A positive result indicates that the avirulent microorganism was inactivated to a higher degree (less resistant) than B. anthracis Ames. *, a significant difference in efficacy; —, no testing under that condition.

## DISCUSSION

### Inoculation and recovery.

The titer of the spore stock solutions for each test was consistent throughout the study, resulting in inoculum levels within the range of 10^8^ CFU per coupon ± 30%. The average percent recovery of spores from positive controls (Table 3) was within our target range of 1 to 150% for all spores with the exception of G. stearothermophilus spores on ceiling tile (average value, 0.4%). The average recovery of spores from the bare pine wood and unpainted concrete positive controls was significantly lower than the average recovery from other materials and ranged from approximately 2 to 13% across all species/strains. The lower recovery of spores from these three materials is most likely due to the porosity of these materials, and the rate of recovery is comparable to that found in previously described tests (13). Nevertheless, spore recovery was still sufficient to quantify an LR of >6.

### Decontamination efficacy.

Decontamination tests were conducted with MeBr at concentrations of 212 mg/liter and 300 mg/liter, temperatures of 22, 27, and 32°C, and RH levels of 45 and 75%, with the goal of determining the minimum contact time required to obtain at least a 6-LR of B. anthracis Ames on all materials for a given fumigation condition. As illustrated in Table 6, these minimum required contact times decreased with increasing concentration and temperature when testing was performed at 75% RH (the effect of these parameters at a low RH is discussed further below). We note that these are the discrete contact times tested and demonstrated to achieve a ≥6-LR of Ames on all materials but that, in actuality, reduced contact times would most likely suffice. For example, under the fumigation conditions using 212 mg/liter MeBr, 27°C, and 75% RH, we report that 36 h was needed to achieve a ≥6-LR on all materials on the basis of the test 9 results, but the actual contact time needed would more likely be between 24 and 36 h, since there was only one material not decontaminated to a ≥6-LR at 24 h (test 7).

Although a few studies in the scientific literature have reported on the sporicidal efficacy of MeBr (8–11), only the U.S. Environmental Protection Agency (11) included experiments that were conducted under conditions similar enough to those of the present study to allow a comparison with our results. For example, Kolb and Schneiter (8) assessed efficacy at 3,400 to 3,900 mg/liter MeBr (more than 10 times the concentrations that we used) with different B. anthracis strains inoculated onto filter paper but did not report the RH level. The maximum MeBr concentration tested against Bacillus megaterium and Bacillus subtilis on membrane filters by Schade and King (9) was 64 mg/liter. Juergensmeyer et al. (10) conducted all tests with spores on glass slides and fumigation with MeBr at 37°C with a 48-h contact time, but they did not measure the RH and used a maximum MeBr level of 112 mg/liter. One test conducted by the U.S. Environmental Protection Agency (11) at 212 mg/liter MeBr, 75% RH, and 25°C showed that all but one material was decontaminated at a level of a ≥6-LR using a 24-h contact time. This result is comparable to that of test 7 of the present study (Table 4), which used the same concentration, contact time, and RH level used in the previous study and a similar temperature (27°C), and all but one material (glass) was decontaminated at a level of a ≥6-LR.

Two RH levels were assessed in the present study for their effect on decontamination efficacy, and the general finding was that efficacy was improved at the higher RH. While several materials were completely decontaminated when fumigation was at 45% RH, there were no tests in which a ≥6-LR was achieved on all materials when fumigation was at 45% RH. Even with increases in the MeBr concentration to 300 mg/liter, the contact time to 72 h, and the temperature to 32°C (not all of which were tested simultaneously, however), we were still unable to obtain a ≥6-LR on all materials when fumigation was at 45% RH. In contrast, there were several test conditions in which a ≥6-LR was achieved on all materials when fumigation was at 75% RH. Additionally, in a comparison of individual tests in which all fumigation conditions except RH were the same, e.g., tests 1 and 3, we show that an increase in the RH from 45% to 75% improves efficacy significantly. The improved decontamination efficacy achieved with higher RH levels is consistent with the findings of Kolb and Schneiter (8), who reported improved efficacy using MeBr with the presence of moisture. Whitney et al. (17) and Davies et al. (18) both discussed several fumigants and other decontamination technologies in which improved inactivation of B. anthracis and Clostridium difficile spores, respectively, occurs at higher RH levels, although neither specifically mentioned MeBr gas as the decontaminant.

Painted wallboard paper and unpainted concrete had significantly higher (at the 10% significance level) LR values for B. anthracis Ames than all other materials except carpet. For this reason, these materials were eliminated from further testing after test 8. As can be seen in Table 4, in nearly every test the Ames strain was completely inactivated on painted wallboard paper and unpainted concrete, including the tests at 45% RH. Conversely, the LR levels for Ames on glass and bare pine wood were significantly lower than those on all other materials.

Just one study found in the scientific literature examined the effect of material on the decontamination efficacy of MeBr (11). Many decontamination studies often overlook the effect of material and may use just one typical material from the laboratory, such as filter paper (8, 9) or glass slides (10). In addition, while glass has been shown to be relatively easy to decontaminate with other sporicidal fumigants, such as hydrogen peroxide vapor (14) and formaldehyde (19), this was not the case in this study. The mechanism of MeBr germicidal activity has been hypothesized by Kolb and Schneiter (8) to be attributed to hydrobromic acid, while Bulathsinghala and Shaw (20) suggested that MeBr's toxicity is due to its high chemical reactivity, leading to the methylation of biological molecules (e.g., DNA alkylation). It may be that the reactivity of MeBr with bacterial spores is diminished by chemical reactions with certain substrate materials, such as glass. However, the glass that we used for the fabrication of the coupons was prepared according to a standard specification (21), and no chemical anomalies that would make the glass particularly reactive with MeBr would be expected.

Three different avirulent spore species were tested for comparison with B. anthracis Ames spores during the study. The first two tests were conducted using G. stearothermophilus spores. All the LR values for G. stearothermophilus spores were higher than those for spores of the Ames strain (average difference, an approximately 1- to 2-LR) for every material in tests 1 and 2, except when both materials were completely decontaminated. It was shown that G. stearothermophilus was less resistant than the B. anthracis Ames strain, and these findings are consistent with those of the five experiments conducted by Juergensmeyer et al. (10), in which G. stearothermophilus was less resistant than the virulent B. anthracis ANR-1 strain. In the present study, the NNR1Δ1 strain was inactivated significantly less than the Ames strain in every trial in which it was used. In the tests conducted at 75% RH, the NNR1Δ1 strain tended to be even more resistant than Ames when the results were compared to the results obtained at 45% RH, with the differences between it and Ames reaching LR values as high as 6.0 (see Table 4, test 3, for example, and Table 8). This observation corresponds with the results obtained for the Sterne strain, in that the Sterne strain was less resistant than Ames for every test at 45% RH but was more resistant than Ames for all MeBr fumigations at 75% RH. Unfortunately, there are no related data in the literature with which to compare our data. These results suggest that an increase in RH improves the inactivation of the Ames strain (via MeBr gas) proportionately more so than that of the Sterne or NNR1Δ1 strain. Further, the results indicate that the NNR1Δ1 strain may be a reasonable surrogate for use in future tests with MeBr when fumigation is at 45% RH, whereas the Sterne strain may be a reasonable surrogate for the Ames strain when tests are performed with MeBr at 75% RH. Further research may be warranted to elucidate any physiological differences of the endospores of the three B. anthracis strains tested, to assist in explaining the differences in their resistance to MeBr. For example, both the NNR1Δ1 (22) and Sterne (23) strains lack the pXO2 plasmid, needed for vegetative cell capsule production, while the Ames strain includes this plasmid and capsule. However, it is acknowledged that the capsular differences in the strains as vegetative cells may be irrelevant in their resistance to chemical treatment as spores.

## Supplementary Material

Supplemental material
