# Supplementary material for: Evaluation of the Efficacy of Methyl Bromide in the Decontamination of Building and Interior Materials Contaminated with Bacillus anthracis Spores
Source: Appl Environ Microbiol. 2016 Mar 21;82(7):2003–11. doi: 10.1128/AEM.03445-15 (PMC4807506; doi:10.1128/AEM.03445-15)
Supplement: Supplemental material [file supp_82_7_2003__index.html]

Supplemental material 

# Evaluation of the Efficacy of Methyl Bromide in the Decontamination of Building and Interior Materials Contaminated with Bacillus anthracis Spores

## Supplemental material

- Supplemental file 1 -

  Actual fumigation conditions for MeBr tests (Table S1).

  PDF, 19K
