# Supplementary material for: Evaluation of the Efficacy of Methyl Bromide in the Decontamination of Building and Interior Materials Contaminated with Bacillus anthracis Spores
Source: Appl Environ Microbiol. 2016 Mar 21;82(7):2003–11. doi: 10.1128/AEM.03445-15 (PMC4807506; doi:10.1128/AEM.03445-15)
Supplement: Supplemental material [file AEM.03445-15_zam999117030so1.pdf]

Supplemental Material

Table S1 Actual fumigation conditions for MeBr tests

Test Number	MeBr Concentration (mg/l)		Temperature (°C)		RH (%)		Contact Time (h)[†]
	Target	Actual*	Target	Actual*	Target	Actual*	
1	212	213.39 ± 5.50	22	22.47 ± 0.31	45	45.03 ± 0.59	36
2	212	211.16 ± 2.95	22	22.20 ± 0.38	45	46.45 ± 1.87	48
3	212	211.74 ± 3.29	22	22.41 ± 0.16	75	75.29 ± 0.27	36
4	212	213.01 ± 4.23	27	27.58 ± 0.51	45	48.66 ± 8.90	36
5	212	212.38 ± 2.87	22	22.13 ± 0.13	75	74.90 ± 0.35	24
6	212	212.16 ± 2.96	27	27.14 ± 0.16	45	45.75 ± 1.63	48
7	212	212.17 ± 2.79	27	27.14 ± 0.23	75	75.98 ± 1.61	24
8	212	212.55 ± 3.14	27	27.13 ± 0.10	45	45.39 ± 1.52	48
9	212	211.97 ± 2.67	27	27.24 ± 0.14	75	75.72 ± 1.56	36
10	300	301.29 ± 3.07	22	22.32 ± 0.13	45	45.81 ± 1.30	48
11	212	210.81 ± 2.72	22	21.99 ± 0.19	45	46.07 ± 1.60	60
12	212	212.78 ± 3.21	32	32.14 ± 0.23	75	75.28 ± 1.64	24
13	212	212.16 ± 2.98	32	32.16 ± 0.16	45	45.99 ± 1.15	48
14	300	302.62 ± 3.49	22	22.20 ± 0.28	75	76.30 ± 2.36	24
15	300	302.87 ± 3.74	22	22.23 ± 0.11	45	46.10 ± 1.19	60
16	212	211.38 ± 2.93	32	32.17 ± 0.29	45	45.79 ± 0.84	60
17	300	300.87 ± 2.76	27	25.56 ± 0.58	75	81.16 ± 2.99	18
18	300	301.18 ± 2.86	27	27.28 ± 0.21	45	45.95 ± 1.03	60
19	212	212.10 ± 3.82	32	32.16 ± 0.28	45	46.23 ± 1.18	72
20	300	301.53 ± 4.41	32	32.14 ± 0.18	45	45.87 0.89	60

*Actual data reported as average ± SD.
[†] Contact time did not deviate from target during any test.
